# Age and sex trend differences in hemoglobin levels in China: a cross-sectional study

**DOI:** 10.1186/s12902-022-01218-w

**Published:** 2023-01-09

**Authors:** Fan Su, Lei Cao, Xia Ren, Jian Hu, Grace Tavengana, Huan Wu, Yumei Zhou, Yuhan Fu, Mingfei Jiang, Yufeng Wen

**Affiliations:** 1 School of Public Health, Wannan Medical College, Anhui Province, No.22, Wenchang Xi Road, Wuhu, 241002 China; 2 School of Laboratory Medicine, Wannan Medical College, Wuhu, Anhui Province, China; 3 School of Clinical Medicine, Wannan Medical College, Wuhu, Anhui Province, China

**Keywords:** Hemoglobin, Age, Changing trend, Gender

## Abstract

**Background:**

Both age and gender are the influence factors of hemoglobin concentration. However, the changing trend of hemoglobin levels between males and females with age remains unclear. This study aimed to explore their changing characteristics in different genders.

**Methods:**

A cross-sectional study was conducted in Physical Examination Center of the First Affiliated Hospital of Wannan Medical College in Wuhu, China from 2014 to 2016. The generalized linear model was applied to explore the relationship between age, gender and hemoglobin levels.

**Results:**

Among the 303,084 participants, the mean age for females and males was 46.9 ± 13.4(15–98) and 48.1 ± 13.7(14–98) years old, respectively. Generalized smoothing splines showed that hemoglobin levels increased up to age 25 and then decreased in men; in women the levels increased up until age 20, and then decreased, with slight increase again (*β* = 0.244, *P* < 0.01). After dividing all participants into hyperglycemia and normal groups, only the normal female group showed a significant upward trend (*β* = 0.257, *P* < 0.01) between ages 50–59.

**Conclusions:**

Hemoglobin concentration changes with age and the curve is different in males and females. The slightly upward trend of female hemoglobin in the age range of 50–59 years old should be considered in developing the reference range of hemoglobin making.

## Background

Hemoglobin is a protein locates in erythrocytes, which accounts for 95% to 97% of the cytosolic protein in erythrocytes, and functions as a transporter that carries oxygen to peripheral tissues [1]. Since its irreplaceable function in life activities, abnormality of hemoglobin can lead to pathological conditions. Anemia is remaining a severe public health problem in developing countries which is mainly caused by low hemoglobin levels. Various diseases have been testified to be associated with anemia, including stroke, coronary artery disease, sickle-cell disease, etc. [2, 3]. Side effects were also found in a higher level of hemoglobin, such as polycythemia, plateau residents and newborns [4–6]. Moreover, mortality and incidence of complications of cardiovascular disease in diabetic patients could be affected by hemoglobin concentration [7]. Collectively, hemoglobin concentration has become an important monitoring indicator of health status. Former scholars mostly studied aspecial periods like childhood, pregnancy and the elderly [8–10]. Besides, researchers have identified hemoglobin concentration could be affected by age, gender, BMI, pregnancy and other factors [11–13], but seldom have studies focused on the changes in hemoglobin concentration with age in different gender yet.

According to World Health Organization (WHO) standards [14], the diagnosis of anemia is only adjusted by sex, children and pregnancy. Therefore, the standard may ignore people in special periods such as the elderly and menopausal women [12]. In this study, we analyzed the relationship between hemoglobin and age in a large-scale health check-up population, and how hemoglobin concentration changes with age in different gender and blood glucose groups. We hope this study can provide a basis for new hemoglobin threshold standards making and relevant disease diagnosis.

## Materials and methods

### Subjects

A cross-sectional study was conducted to investigate how hemoglobin concentration changes with age. All participants were enrolled from the Physical Examination Center of the First Affiliated Hospital of Wannan Medical College in Wuhu, China, from 2014 to 2016. The exclusion criteria included: (1) absence of available data on triglyceride (TG), fasting blood glucose, uric acid, high-density lipoprotein, urea nitrogen, glutting-pyruvic transaminase, glutamic-oxaloacetate, aminotransferase, hemoglobin; (2) individuals with severe brain disease intervention, tumor or cancer, severe cardiovascular diseases, or severe infections. A total of 303,084 participants made of 176,614 (58.3%) males and 126,470 (41.7%) females had undergone a health check upon request. Their mean age was 47.6 ± 13.6 (10–98) years. The study was conducted in compliance with Helsinki guidelines of the Helsinki Declaration of the World Medical Association and approved by the Ethics Committee of Wannan Medical College. Verbal informed consent was obtained from each participant before the investigation.

### Questionnaire survey

The questionnaire was designed by experts in epidemiology and clinical doctors. Demographic and behavioral characteristics, history of diseases and operations, and body examinations were included in the questionnaire. Demographic characteristics included age, sex, and occupation. Smoking (never smoking: never smoking in the past year; smoking occasionally: smoking more than 1 day but less than 3 days a week; smoking frequently: smoking more than 3 days a week), and drinking alcohol (never drinking: never drinking in the past year; drinking occasionally: drinking more than 1 time but less than 3 times a week; drinking frequently: drinking more than 3 times a week) was classified as behavioral characteristics. Information about severe infections, cardiovascular diseases, major surgeries, medication, and cancer was contained in the column of history of the disease.

### Physical examination

Physical examination was conducted by professionals following the Hypertension guidelines of World Health Organization/International Society [15]. Blood pressure was measured using a mercury sphygmomanometer after the subjects rested for 5 min. Body mass index was calculated by weight (kg)/square of height (m^2^). Data of BMI were calculated to the nearest 0.01 kg/m^2^.

### Biochemical assays

Fasting venous blood was collected in the morning and related indicators were detected. Parameters had been detected included fasting blood glucose (FBG), triglyceride (TG), cholesterol (TC), high-density lipoprotein cholesterol (HDL-C), glutamic-pyruvic transaminase enzyme (ALT), glutamic-oxaloacetic aminotransferase (AST), hemoglobin (HGB), creatinine (Cre). All biochemical assays were performed by professionals in the hospital.

### Definitions

Hypertension (HBP) was defined as systolic pressure (SBP) ≥ 140 mmHg or diastolic pressure (DBP) ≥ 90 mmHg. Hyperglycemia was defined as fasting blood glucose ≥ 6.1 mmol/L or hypoglycemic drugs that were currently being taken [16]. Hyperlipidemia was defined as TG ≥ 2.3 mmol/L or HDL-C < 0.9 mmol/L or blood lipid medication was conducted currently. Obesity was defined as BMI ≥ 25 kg/m^2^.

### Statistical analysis

Data were expressed as mean ± SD and frequency (%). Differences between men and women with indicators were compared through t test or chi-square test. Generalized smoothing spline was used to analyze the possible nonlinear relationship between hemoglobin and age, and the knot locations was generated automatically in generalized additive models with R package MGCV. The different divide of age periods were based on the knot locations. Linear regression analysis was conducted to analyze the relationship between hemoglobin and different age periods. All data were analyzed by SPSS 18.0 and R software program (V.3.0.0).

## Results

### Characteristics of subjects

Table 1 showed that the mean age was 46.9 ± 13.4 (15–98) years old in females, and 48.1 ± 13.7 (14–98) years old in males. The concentration of hemoglobin in male was higher than female group (*P* < 0.01). Besides, FPG, TC, TG, AST, ALT, Cre, BMI, and blood pressure level were higher among males (*P* < 0.01). Significant differences in other demographic characteristics and biochemical indicators were also observed between the two gender groups (Table 1).

**Table 1:** Comparison of characteristics of male and female group

Variables	Female (*n* = 126,470)	Male (*n* = 176,614)	*P*
Age(year)	46.9 ± 13.4	48.1 ± 13.7	< 0.01
BMI(kg/m^2^)	22.8 ± 3.2	24.5 ± 3.1	< 0.01
SBP(mmHg)	115.4 ± 17.0	122.1 ± 16.2	< 0.01
DBP(mmHg)	74.2 ± 9.2	79.7 ± 9.8	< 0.01
FBG(mmol/l)	5.3 ± 0.9	5.5 ± 1.3	< 0.01
TG(mmol/l)	1.3 ± 0.9	1.8 ± 1.4	< 0.01
TC(mmol/l)	4.6 ± 0.9	4.7 ± 0.9	< 0.01
HDL-C(mmol/l)	1.5 ± 0.4	1.3 ± 0.3	< 0.01
AST(U/L)	20.5 ± 10.8	25.4 ± 16.2	< 0.01
ALT(U/L)	19.3 ± 16.5	33.0 ± 30.0	< 0.01
HGB(g/L)	127.7 ± 11.3	150.2 ± 11.2	< 0.01
Cre(μmol/l)	56.8 ± 13.6	79.6 ± 18.0	< 0.01
Drink			
Never	98.7%	43.7%	< 0.01
Occasionally	0.1%	16.2%	< 0.01
Frequently	1.2%	40.1%	< 0.01
Smoke			
Never	99.8%	53.7%	< 0.01
Occasionally	0.1%	36.9%	< 0.01
Frequently	0.1%	9.4%	< 0.01
Obesity			
No	78.4%	57.2%	< 0.01
Yes	21.6%	42.8%	< 0.01
HBP			
No	87.6%	76.6%	< 0.01
Yes	12.4%	23.4%	< 0.01
Hyperglycemia			
No	91.7%	85.2%	< 0.01
Yes	8.3%	14.8%	< 0.01
Hyperlipidemia			
No	81.5%	57.8%	< 0.01
Yes	18.5%	42.2%	

^*^BMI body massive index, *SBP* systolic blood pressure, *DBP* diastolic blood pressure, *FBG* fasting blood glucose, *TG* triglyceride, *TC* total cholesterol, *HDL-C* high density liptein cholesterol, *AST* aspartate aminotransferase, *ALT* glutamic-oxaloacetic aminotransferase, *HGB* hemoglobin, *Cre* creatinine

### Sex-specific analysis of the relationship between hemoglobin and age

**Fig. 1:**
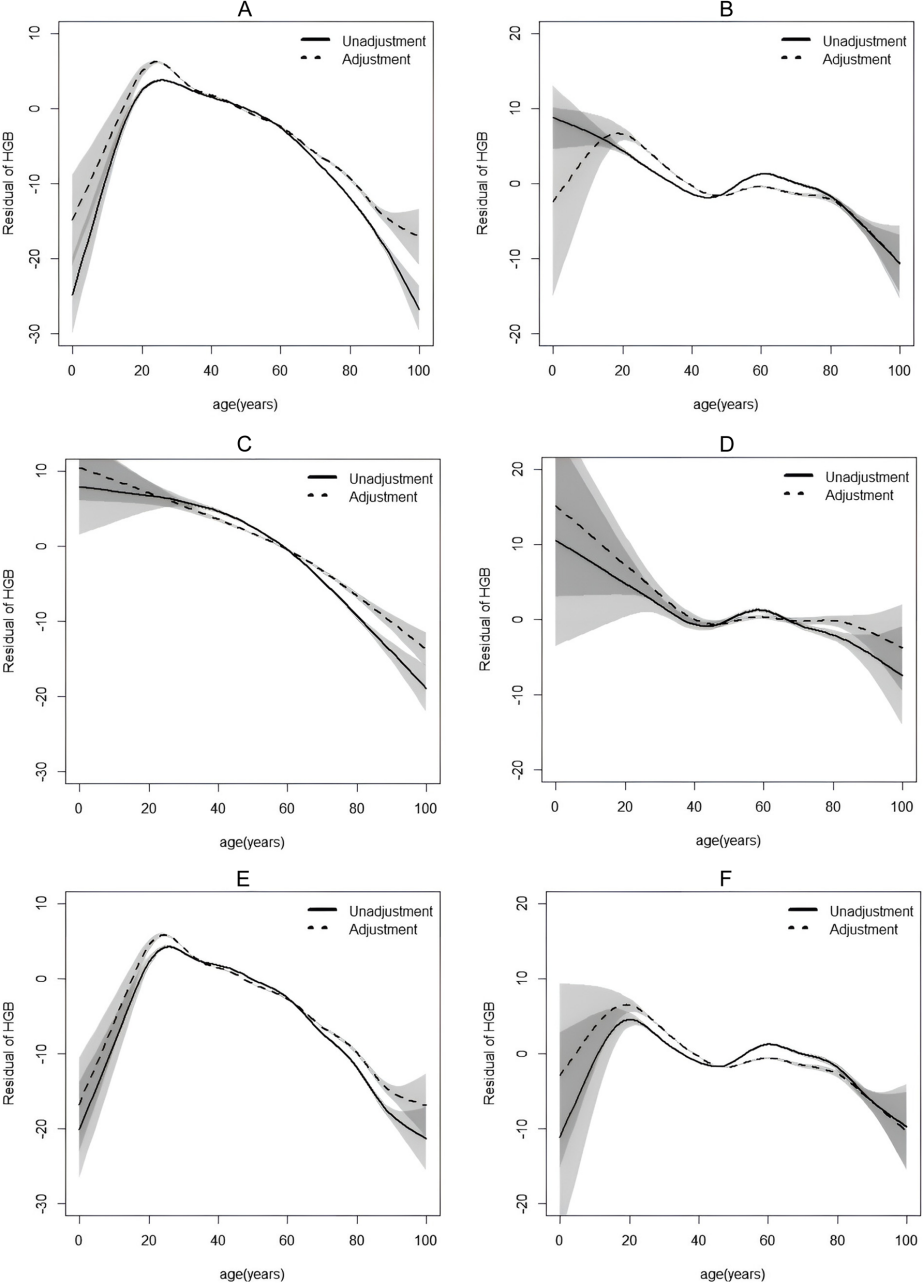
Relationship between hemoglobin concentration and age (The horizontal coordinate represents age; the longitudinal coordinate shows residual of HGB. Solid line: without adjustment; dotted line adjustment for drink, smoke, BMI, SBP, DBP, FBG, TG, TC, HDL-C, ALT, AST, Cre. Shaded area represents confidence interval. **A** Relationship between hemoglobin concentration and age in males. **B** Relationship between hemoglobin concentration and age in females. **C** The curve of hemoglobin with age in hyperglycemia group of males. **D** The curve of hemoglobin with age in hyperglycemia group of females. **E** The curve of hemoglobin with age in non-hyperglycemia group of males. **F** The curve of hemoglobin with age in non-hyperglycemia group of females)

Specific analysis was performed on men and women respectively. In male group, hemoglobin levels increased with age before 25 years old, then the curve showed a downward trend (Fig. 1A, Table 2). Hemoglobin levels increased when females were younger than 20 years old and decreased then, while a slight upward trend in hemoglobin levels was observed in women aged 50–60 years old (Fig. 1B and Table 3).

**Table 2:** Relationships between hemoglobin and age according to blood glucose in males

Age (years)	Ungrouped			Hyperglycemia^a^			Non-Hyperglycemia		
	*β*	SE	*P*	*β*	SE	*P*	*β*	SE	*P*
< 25	0.614	0.147	< 0.01	-0.196	0.003	< 0.01	0.615	0.143	< 0.01
≥ 25	-0.264	0.002	< 0.01	-0.293	0.008	< 0.01	-0.262	0.002	< 0.01

β stands for coefficient of age in generalized liner model, *SE* stands for standard deviation

^a^From Fig. 1C, there was no Inflection point of age

**Table 3:** Relationships between hemoglobin and age according to blood glucose in females

Age (years)	Ungrouped			Hyperglycemia			Non-hyperglycemia		
	*β*	SE	*P*	*β*	SE	*P*	*β*	SE	*P*
< 50	-0.284	0.006	< 0.01	-0.181	0.043	< 0.01	-0.286	0.006	< 0.01
50–59	0.244	0.028	< 0.01	0.102	0.081	0.21	0.257	0.030	< 0.01
≥ 60	-0.121	0.010	< 0.01	-0.078	0.021	< 0.01	-0.138	0.011	< 0.01

β stands for coefficient of age in generalized liner mode, *SE* stands for standard deviation

### Relationship between hemoglobin with age in different blood glucose groups

After dividing all participants into hyperglycemia and normal group, the hemoglobin concentration decreased throughout all age in hyperglycemia group of males (*β* = -0.270, *P* < 0.01) (Fig. 1C). In the hyperglycemia group of females (Fig. 1D, Table 3), the curve had been declining until 50 years old (*β* = -0.181, *P* < 0.01), but the tiny rise between 50 and 60 years old showed no significance (*β* = 0.102, *P* = 0.21), then it declined after 60 years old (*β* = -0.078*, P* < 0.01). In the non-hyperglycemia group, the trend of curve was basically consistent with that before grouping (Fig. 1E and F).

## Discussion

Determining the changing trend of hemoglobin levels by age and sex has important clinical uses. Understanding where natural fluctuations occur can help determine anemia in different populations, such as menopausal women and the elderly, or the effects of cancer chemotherapy [17, 18], or aid development of effective nutritional strategies for athletes as well as the general population.

In all participants, hemoglobin levels of both genders showed an overall downward trend with age after reaching the peak in both gender. Some previous studies reported hemoglobin continues to increase with age before reaching a plateau among teenagers [19], our results were consistent with these findings. With the improvement of living material level, the incidence of adolescence anemia due to iron deficiency is greatly reduced, hemoglobin showed an upward trend as the body gradually develops and matures [20, 21]. Song W [22] reported similar results that hemoglobin peaks earlier in adolescent females than in males, periodic blood loss in females may be the cause of this result [23]. Hemoglobin concentration started to decrease with age after reaching the peak. Hemoglobin decreased with age in male subjects, and this decrease may be due to a progressive loss of androgens, as androgens stimulate increased production of red blood cells [24]. In females, the decreasing tendency was also reported by Taneri PE [25], this could be due to reduction in expression of androgens with age, reduced iron intake from weight loss and blood loss during menstruation [26, 27]. Most females would experience menopause around 42 ~ 58 years old, the decrease in blood loss led to a brief rise in their hemoglobin levels [28]. Due to the degeneration of organ function, malnutrition, low immune function, secondary to other systemic diseases or even malignant tumors, the elderly are a high-risk group for anemia, and hemoglobin decreases with age [29].

After grouping participants into hyperglycemia and normal group, we had drawn two similar J-shaped curves of males and females in hyperglycemia group (Fig. 1C-D). There was a slight difference that the rising trend in adolescence was gone in both hyperglycemia groups, mainly caused by the lack of subjects of the young. It was interesting that the coefficient of the 50 ~ 60 years old section showed no statistical significance in women. Hemoglobin concentrations in postmenopausal women usually increase moderately, and it could irreversibly bind with glucose to form glycosylated hemoglobin when blood glucose levels increased [30, 31]. Chronic hyperglycemia causes excessive production or accumulation of reactive oxygen species (ROS), and directly activates calcium-sensitive K + channels. Also, KCl leaks out of the cells with the cytosol, resulting in a decrease in cell volume and loss of membrane integrity, which leads to the onset of erythrocyte death, and consequently a decrease in hemoglobin [32, 33]. For hyperglycemia women of the 50 ~ 60 years old, the meaningless upward trend may be due to a decrease in the hemoglobin concentration that should have been elevated. In the non-hyperglycemic group, the trend in hemoglobin with age was similar to the overall (Fig. 1D, E, Tables 1 and 2).

In this study, the results showed that the trend of hemoglobin levels with age is different between male and female, female's unique menstrual cycle plays a crucial role in this difference. High blood glucose levels can also have an effect on hemoglobin. Consequently, it is important to consider these above factors when determining the hemoglobin reference intervals and criteria for anemia in different populations. We conducted the study based on a cross-sectional study, and due to the limitations of this method, we only analyzed the results at one time point and failed to provide a continuous observation of the study population. However, we observed the effect of various factors on hemoglobin levels. Women during pregnancy were not included in this study, and further data will be collected in the future to develop more comprehensive results.

## Conclusion

Hemoglobin concentration changed with age and showed different curve in men and women. Results obtained from the study indicated the referenced range of hemoglobin should consider the age and gender effect.

## Data Availability

The datasets generated during the current study are not publicly available due to the data used in this study was under license from the Health Management Center at the First Afliated Hospital of Wannan Medical College for the current study, but are available from the corresponding author on reasonable request with permission from the Health Management Centre of the First Afliated Hospital of Wannan Medical College.
